# Rise-Time of FRET-Acceptor Fluorescence Tracks Protein Folding

**DOI:** 10.3390/ijms151223836

**Published:** 2014-12-19

**Authors:** Simon Lindhoud, Adrie H. Westphal, Carlo P. M. van Mierlo, Antonie J. W. G. Visser, Jan Willem Borst

**Affiliations:** 1 Laboratory of Biochemistry, Wageningen University, Wageningen 6703HA, The Netherlands; E-Mails: s.lindhoud@tudelft.nl (S.L.); adrie.westphal@wur.nl (A.H.W.); carlo.vanmierlo@wur.nl (C.P.M.M.); antoniejvisser@gmail.com (A.J.W.G.V.); 2 Department of Bionanoscience, Kavli Institute of Nanoscience, Delft University of Technology, Delft 2628CJ, The Netherlands; 3 Microspectroscopy Centre, Wageningen University, Wageningen 6703HA, The Netherlands

**Keywords:** time-resolved fluorescence, protein folding, Alexa Fluor, FRET, rise time of acceptor fluorescence

## Abstract

Uniform labeling of proteins with fluorescent donor and acceptor dyes with an equimolar ratio is paramount for accurate determination of Förster resonance energy transfer (FRET) efficiencies. In practice, however, the labeled protein population contains donor-labeled molecules that have no corresponding acceptor. These FRET-inactive donors contaminate the donor fluorescence signal, which leads to underestimation of FRET efficiencies in conventional fluorescence intensity and lifetime-based FRET experiments. Such contamination is avoided if FRET efficiencies are extracted from the rise time of acceptor fluorescence upon donor excitation. The reciprocal value of the rise time of acceptor fluorescence is equal to the decay rate of the FRET-active donor fluorescence. Here, we have determined rise times of sensitized acceptor fluorescence to study the folding of double-labeled apoflavodoxin molecules and show that this approach tracks the characteristics of apoflavodoxinʼs complex folding pathway.

## 1. Introduction

Over the past few decades, Förster resonance energy transfer (FRET) has become a popular tool to probe distances between fluorescent donor and acceptor molecules. FRET is often employed to verify or refute dynamic interactions between proteins, both *in vitro* and *in vivo* [1,2,3,4]. In addition, FRET is used to study conformational changes within proteins that contain donor and acceptor fluorophores, for instance upon binding of ligands or substrates. FRET is the transfer of electronic excitation energy from an excited donor fluorophore to an acceptor chromophore in the ground state through non-radiative dipole-dipole coupling [5,6]. The rate constant of FRET (*k*_t_) depends on the inverse sixth power of the distance between the donor and acceptor, and FRET efficiency measurements can therefore be exploited as a spectroscopic nanometric ruler.

FRET measurements are greatly facilitated by the use of brightly fluorescent dyes that emit and absorb in the visible spectrum, such as Alexa Fluor dyes, Atto dyes, BODIPY dyes and cyanine dyes [7]. Labeling of proteins with donor and acceptor dyes is preferably site-specific, because the fluorescent probes commonly used are sensitive to changes in their local environments. However, homogeneous and site-specific double labeling of proteins with fluorophores is often challenging and not straightforward (see, for instance, [8,9,10]). When preparing fluorescently labeled protein variants for FRET experiments, one would like to minimize the costly and time-consuming efforts to devise site-specific labeling strategies for each putative FRET-sensor. Therefore, it would be advantageous to have a method to screen whether a certain preparation of double-labeled proteins displays a change in FRET efficiency upon interaction with a certain stimulus (e.g., titration with a ligand and an interacting protein). However, protein preparations that are double-labeled with donor and acceptor to non-equimolar ratios lead to inaccurate determination of FRET values. Particularly, protein molecules that contain only donor fluorophores and, thus, emit more donor photons compared to molecules containing both labels cause an underestimation of the actual FRET efficiency [11].

In this study, we overcome the limitations of FRET determination in ensemble measurements as posed above, by determining the arrival times of acceptor photons upon pulsed excitation of a fluorescent donor. Thus, we determine the “rise time” of acceptor fluorescence, which can be used to quantify the rate of transfer of excited state energy from the donor to acceptor [12,13,14,15,16]. We have performed time-resolved measurements of sensitized acceptor fluorescence to study the denaturant-dependent folding of apoflavodoxin from *Azotobacter vinelandii*. This protein is labeled with donor fluorophore Alexa Fluor 488 C5 maleimide (A488) and acceptor fluorophore Alexa Fluor 568 C5 maleimide (A568). These dyes are brightly fluorescent, having a high fluorescence quantum yield (>0.5), are relatively photostable and can be excited with visible light [7,17]. A488 is often used as a donor fluorophore in single-molecule FRET experiments (see, e.g., [18,19,20,21]) and in protein ensemble measurements (see, e.g., [22,23]).

Both equilibrium and kinetic (un)folding of apoflavodoxin have been characterized using guanidine hydrochloride (GuHCl) as the denaturant (see, for instance, [24,25]. Equilibrium folding of apoflavodoxin can be described by the three-state model *N*↔*U*↔*I*_off_, in which *N* and *U* are native and unfolded protein, respectively, and *I*_off_ is an intermediate that is kinetically off-pathway [24]. This intermediate has molten globule-like properties and populates to significant extents at denaturant concentrations ranging from about 1 to 3 M GuHCl. In this folding species, both native and non-native α-helices form and dock onto each other in a non-native-like fashion [26,27,28].

Previously, we demonstrated that folding of apoflavodoxin could be tracked using steady-state fluorescence on molecules that were site-specifically labeled with A488 and A568 [22]. Here, we test the potential of the rise time of acceptor fluorescence to probe folding of double dye-labeled apoflavodoxin. The rise time of acceptor fluorescence is equivalent to donor fluorescence lifetime in the presence of an acceptor (τ_da_) [14,15,16] (see Section 3.1 for the theoretical background). The key advantage of this spectroscopic approach is that one isolates single, FRET-active pairs within a heterogeneous population of fluorophore-labeled proteins. We show that measurement of the rise time of acceptor fluorescence reveals folding-induced conformational changes of apoflavodoxin and, thus, tracks protein folding.

## 2. Results and Discussion

### 2.1. Acceptor Rise and Decay Times and Their Corresponding Amplitudes Track (Un)folding of Apoflavodoxin

**Figure 1:**
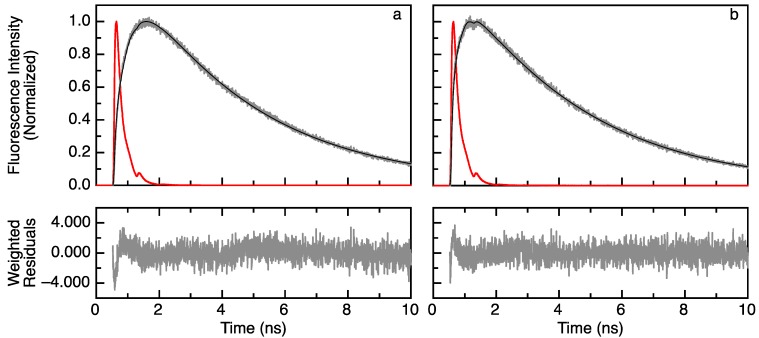
Fluorescence lifetimes with corresponding negative and positive amplitudes characterize the time-resolved fluorescence of the acceptor upon donor excitation. (**Top**) The time dependence of experimental (grey) and fitted (black) fluorescence intensity of apoflavodoxin labeled with A488 (donor) and A568 (acceptor) (see the Experimental Section for details on the theoretical background, protein labeling and data acquisition and analysis). The decay of reference compound erythrosine B has a fluorescence lifetime of 89 ps (red line); (**Bottom**) The weighted residuals between experimental and fitted curves (grey lines). (**a**) Acceptor fluorescence of folded, double-labeled apoflavodoxin in 0 M GuHCl. The rise time is 0.51 ns (confidence limits at the 0.67 confidence level are 0.50 and 0.53 ns); decay time is 3.85 ns (confidence limits at the 0.67 confidence level are 3.84 and 3.86 ns). The absolute value of the amplitude ratio |*A*_−_/*A*_+_| is 0.44. The fit quality criterion χ*^2^* is 1.226; and (**b**) Acceptor fluorescence of unfolded, double-labeled apoflavodoxin in 4.12 M GuHCl. The rise time is 1.57 ns (confidence limits at the 0.67 confidence level are 1.41 and 1.78 ns); decay time is 3.71 ns (confidence limits at the 0.67 confidence level are 3.67 and 3.73 ns). The absolute value of the amplitude ratio |*A*_−_/*A*_+_| is 0.134. The fit quality criterion χ*^2^* is 1.085.

Figure 1 shows examples of experimental and fitted rise and decay curves of apoflavodoxin labeled with donor A488 and acceptor A568. Figure 1a presents the data of folded apoflavodoxin in buffer without denaturant, whereas Figure 1b shows those of unfolded protein in 4.12 M GuHCl. For comparison, the fluorescence response curve of the reference compound, erythrosine B, in water is also shown. Fitting the experimental curve using a model containing a single rise time (with negative amplitude) and a single decay time (with positive amplitude) to the data (as described in Section 3.4) is sufficient, because the fitting criterion χ^2^ is close to the limiting value of one (see the legend in Figure 1) and the weighted residuals randomly fluctuate around zero.

Acceptor fluorescence rise time (τ_da_; Equation (2)) of folded apoflavodoxin is 0.51 ns at 0 M GuHCl, and 1.57 ns in the case of unfolded protein at 4.12 M GuHCl. The corresponding decay times of acceptor fluorescence (τ_a_ = 1/*k*_a_; Equation (2)) are 3.85 and 3.71 ns, respectively. The positions of maximum intensity of fluorescence decay curves of erythrosine B and double-labeled apoflavodoxin differ by 0.94 ns for folded protein, whereas they differ by only 0.76 ns for unfolded protein. This apparent contradiction can be explained by taking into account the ratio between corresponding pre-exponential factors of Equation (3) (*A*_−_/*A*_+_). *A*_−_/*A*_+_ equals −0.443 for folded protein at 0 M denaturant, and *A*_−_/*A*_+_ equals −0.134 for unfolded protein at 4.12 M GuHCl.

**Figure 2:**
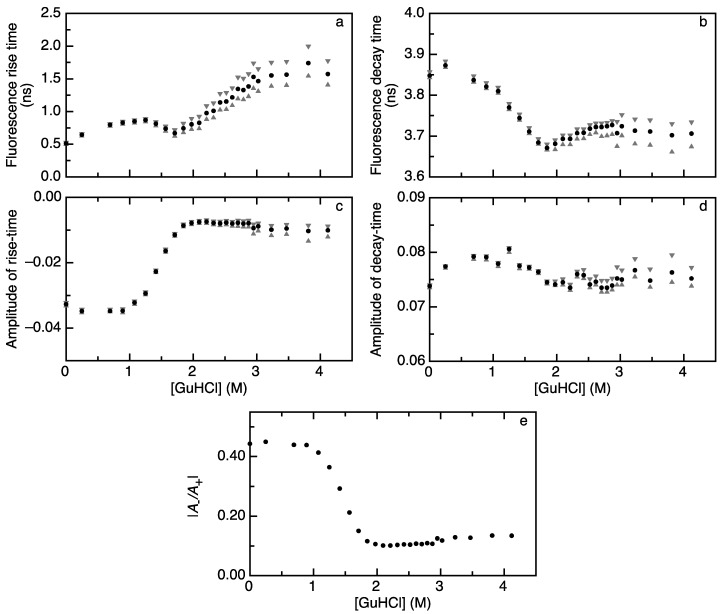
Denaturant-dependencies of rise and decay times of acceptor fluorescence upon donor excitation track folding of apoflavodoxin labeled with A488 (donor) and A568 (acceptor). In all panels, black dots represent fitted values and grey triangles represent confidence limits. (**a**) The rise time of acceptor fluorescence and (**b**) decay time of acceptor fluorescence reveal the biphasic dependencies on GuHCl; (**c**) The amplitude of the acceptor fluorescence rise time (*A*_−_) changes in a monophasic manner as a function of GuHCl; (**d**) The amplitude of acceptor fluorescence decay (*A*_+_) is virtually constant as a function of denaturant concentration; and (**e**) The absolute ratio of the amplitudes of fluorescence rise and decay time (|*A*_−_/*A*_+_|) shows a monophasic dependence on denaturant concentration.

Figure 2 presents the denaturant dependence of rise and decay times of acceptor fluorescence and the corresponding pre-exponential factors. The rise time of acceptor fluorescence (Figure 2a) shows a biphasic dependence on denaturant concentration. Upon going from 0 to 1 M GuHCl, the rise time increases. Subsequently, it displays a minimum at about 1.8 M GuHCl and increases progressively until about 3 M GuHCl. Above this concentration, the rise time remains constant. Such a biphasic folding curve hallmarks the population of a folding intermediate. Indeed, the denaturant dependence of the rise time of acceptor fluorescence reveals the population of apoflavodoxin’s molten globule state. The fluorescence lifetime of the acceptor displays a biphasic transition as a function of denaturant concentration, but the variation is only between 3.9 and 3.7 ns (Figure 2b). This long decay time is comparable to the fluorescence lifetime of the acceptor that is obtained from decay of the acceptor upon direct excitation of the acceptor (Supplementary Information SI1).

Figure 2c,d shows the amplitudes corresponding to the rise and decay times of acceptor fluorescence, respectively. The amplitude of the acceptor fluorescence rise time (*A*_−_) shows a transition between 1 and 2 M GuHCl, whereas the amplitude of the acceptor fluorescence decay time (*A*_+_) does not. Figure 2e shows the absolute ratios of both amplitudes (|*A*_−_/*A*_+_|), which, in contrast to the fluorescence lifetimes, change in a monophasic fashion as a function of denaturant concentration. |*A*_−_/*A*_+_| is constant between 0 and 1 M GuHCl, where apoflavodoxin is native [8,24], and shows a single transition to a lower value upon increasing the denaturant concentration to about 2 M GuHCl. Upon further addition of denaturant, the amplitude ratio remains constant. The transition monitored by the altering amplitude ratio coincides with the denaturant-dependent loss of native apoflavodoxin molecules [8,24] and, thus, tracks the de-population of the native state upon increasing the denaturant concentration.

Based on the value of |*A*_−_/*A*_+_| at low denaturant concentrations, we estimate that 44% of the detected photons arise from sensitized acceptor fluorescence. Hence, the remaining 56% of the detected photons (with positive decay amplitude) arise from other sources (*i.e.*, detection of donor photons and/or direct excitation of the acceptor; see Section 3.1). A decrease in |*A*_−_/*A*_+_| implies that, relative to sensitized acceptor emission, more photons from these other sources are detected. As the FRET efficiency decreases upon unfolding of apoflavodoxin, the fluorescence intensity of sensitized acceptor emission diminishes. Our preparation of labeled protein consists of a population that is labeled with both a donor and acceptor, but also contains molecules that are labeled with only a donor and only an acceptor. If the fluorescence intensities of these latter two molecules are less dependent on the folding state than the fluorescence intensity of sensitized acceptor emission, the ratio between these intensities (reflected by |*A*_−_/*A*_+_|) changes. We previously observed that the folding state does affect both the absorption spectrum, as well as the fluorescence intensity of the fluorophores used [8,22]. These effects are likely caused by the interaction between the dyes and tryptophan residues within the protein and therefore depend on the position of the dye on the protein. Because of these effects, it is highly probable that the amount of photons with a positive amplitude varies as a function of denaturant concentration and, hence, alters |*A*_−_/*A*_+_| further than what can be expected from a change in the intensity of sensitized acceptor emission. The observed decrease in |*A*_−_/*A*_+_| (Figure 2e) is responsible for the observed increase in the confidence limits of the time constants (Figure 2a,b). Another factor to consider is that the rise time becomes longer at higher denaturant concentration. This will also increase the confidence limits, because rise time and decay time will show a higher correlation.

Detailed characterization of the FRET system to elucidate the contributions of each of the effects listed above requires global analysis of donor and acceptor decays, while accounting for the emission of macromolecules labeled with only donor for the donor emission and direct excitation of the acceptor for the double-labeled macromolecules. Such elaborate analysis has been done for other FRET systems, such as pyrene-dendronized porphyrins [29,30], and for the calcium indicator, yellow cameleon [16]. It is worth noting that the rise time of acceptor fluorescence, which contains information about the FRET efficiency, is recovered with good accuracy, even when the absolute amplitude ratio is significantly smaller than unity.

In summary, parameters extracted from fitting the bi-exponential model of Equation (3) to acceptor rise and decay data of double-labeled apoflavodoxin track protein folding.

### 2.2. FRET Rates Derived from Acceptor Rise Times Reveal Conformational Changes during Protein (Un)folding

The rise time of acceptor fluorescence upon donor excitation is equivalent to the donor fluorescence lifetime in the presence of acceptor (τ_da_; Equation (4); see Figure 2a). The transfer rate constant *k*_t_ is obtained from the difference between the reciprocal rise time of the acceptor fluorescence (1/τ_da_) and the reciprocal value of the amplitude-averaged donor fluorescence lifetime in the absence of acceptor (1/τ_d_) (see Equation (5)) [6]. Values of τ_d_ for the denaturant concentrations used in this study were obtained by fitting a three-state model for protein folding to τ_d_ determined previously (see the Supplementary Information SI2). The average fluorescence lifetime of donor-only labeled apoflavodoxin decreases slightly upon unfolding due to changes in dynamic quenching [8].

**Figure 3:**
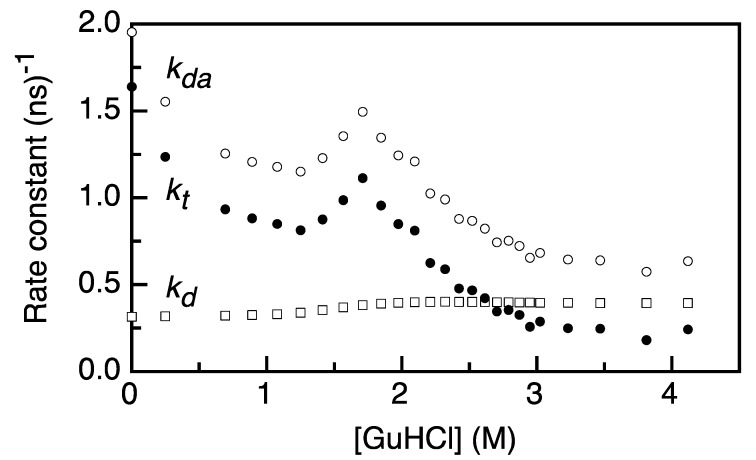
The rate constants of energy transfer change as a function of denaturant concentration. Rate constants are obtained through the relation *k_i_* = 1/τ_i_. *k*_da_ is the decay rate of the donor fluorescence in presence of an acceptor, as obtained from the acceptor rise time (open circles), *k*_d_ is the decay rate of the donor in the absence of an acceptor (open squares) and is obtained from [8], as described in Supplementary Information SI2, and *k*_t_ is the rate of energy transfer, as obtained from the relation *k*_t_ = *k*_da_ *− k*_d_ (Equation (5); black dots).

Figure 3 presents the denaturant-dependent fluorescence decay rates of donor in the absence of acceptor (*k*_d_) and of the fluorescence decay rate of a donor in the presence of an acceptor, as obtained from the rise time of acceptor fluorescence (*k*_da_ = 1*/*τ_da_). We determine the FRET transfer rate constant (*k*_t_) as a function of denaturant concentration by using Equation (5) (Figure 3). *k*_t_ is 1.6 (ns)^−1^ for protein without denaturant. This rate constant decreases to 0.8 (ns)^−1^ upon increasing the concentration of GuHCl to about 1.3 M. *k*_t_ increases upon further addition of denaturant, until it reaches a value of 1.1 (ns)^−1^ at ~1.7 M GuHCl. Subsequently, *k*_t_ gradually decreases, until it levels off to a value of about 0.25 (ns)^−1^ when the denaturant is added to 3 M GuHCl and above. This decrease in *k*_t_ upon the addition of denaturant hallmarks the increase in the distance between the donor and acceptor as apoflavodoxin unfolds.

**Figure 4:**
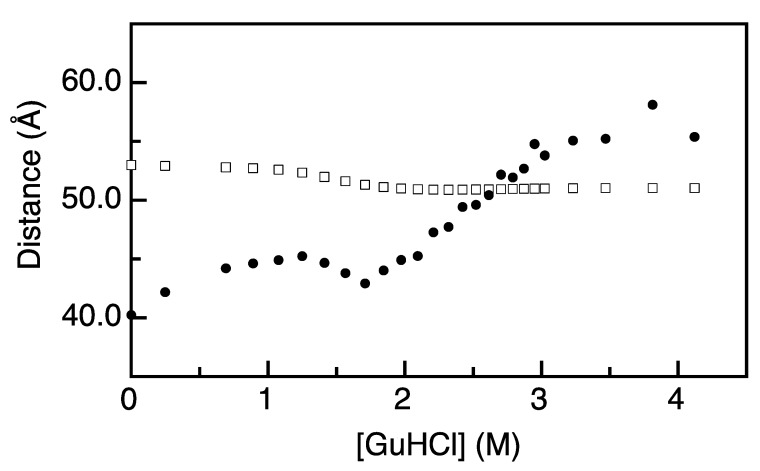
The distance between donor and acceptor fluorophores changes as a function of denaturant concentration and reveals conformational changes during apoflavodoxin (un)folding. The Förster transfer distance (*R*_0_; open squares) changes as a function of denaturant concentration in a folding state-dependent manner. Calculated distances (*r*_da_; black dots) between donor Alexa Fluor 488 and acceptor Alexa Fluor 568 track the features of apoflavodoxin (un)folding. Values of *R*_0_ are obtained by using parameters as described in Supplementary Information SI3. Values of *r*_da_ are obtained by using Equation (7).

The distance *r*_da_ between donor and acceptor labels attached to apoflavodoxin can be estimated from the values of τ_d_ and *k*_t_ via Equation (7). Determination of distances using *k*_t_ and τ_d_ or from FRET efficiencies requires accurate determination of the Förster radius, *R*_0_. Previously, we showed that in the case of apoflavodoxin, *R*_0_ changes as a function of denaturant concentration in a folding state-dependent manner [22]. We recapitulate the corresponding analysis in the Supplementary Information (Supplementary Information SI3) and present the dependence of the inter-dye and Förster distances on the GuHCl concentration in Figure 4.

We determine a distance between donor and acceptor (*r*_da_) of 40 Å for folded apoflavodoxin without denaturant (Figure 4). This distance becomes larger than *R*_0_ upon unfolding of the protein and increases to 55 Å at 4.12 M GuHCl. Each inter-dye distance *r*_da_ must arise from a distribution of fluorophore positions, because both fluorophores reorient on the sub-nanosecond timescale [22]. In the case of unfolded protein, the flexibility of the protein backbone is an additional source of heterogeneity in dye position.

Determination of inter-dye distances that characterize apoflavodoxin’s folding species at each denaturant concentration is not trivial, because the determined rate constants and calculated distances are subject to ensemble averaging. For instance, the population of the native state is approximately 50% at 1.2 M GuHCl and a considerable population of the molten globule and unfolded state exist [8]. However, we derive only a single value for *r*_da_ at this denaturant concentration (Figure 4), which must be considered as an ensemble-averaged distance. Upon increasing the denaturant concentration from 1.2 to about 1.8 M GuHCl, the population of native molecules decreases further, and the molten globular state becomes more populated. Concomitantly, the ensemble-averaged distance between donor and acceptor decreases (Figure 4). Because of this ensemble averaging, we restrict ourselves to qualitative interpretation of the time-resolved fluorescence data. In the denaturant range of 1.2 to 1.8 M GuHCl, the distance between the donor and acceptor is shorter in the molten globule than it is in native protein (Figure 4). The same observation was made using steady-state fluorescence to study the folding of several site-specifically double-labeled apoflavodoxin molecules [22]. Both observations highlight that apoflavodoxin’s molten globule differs considerably from native protein, because in the molten globule, both native and non-native α-helices interact and dock onto each other in a non-native fashion [26].

## 3. Experimental Section

### 3.1. Theoretical and Practical Considerations of Time-Dependent Increase of Acceptor Fluorescence

Suppose we have a homogeneous donor-acceptor system that exhibits FRET from light-excited donor fluorophores to acceptor fluorophores in the ground state and that does not contain donor labels that are FRET-inactive (*i.e.*, that lack an acceptor in their vicinity). Furthermore, we assume that the fluorescence decays of both donor and acceptor are single-exponential, and we excite the donor fluorophores at a wavelength where only donor molecules absorb light, thereby avoiding direct excitation of acceptor molecules. In such a system, we can investigate two time-resolved fluorescence experiments, namely the fluorescence decay of the donor at the maximum emission wavelength of the donor fluorescence spectrum and the time-dependent increase (rise) of the acceptor fluorescence measured at a wavelength where no donor fluorescence photons are detected. Following the solution of differential equations [14], the decay of the excited state concentration of donor (D^*^(*t*)) is directly proportional to the time-resolved fluorescence intensity *I*_DD_(*t*), in which the first subscript *D* denotes donor excitation wavelength and the second subscript *D* denotes donor emission wavelength:

(1) $$\begin{array}{l} D^{*}\left(t\right)=D_{0}^{*}e^{-\left(k_{d}+k_{t}\right)t}\\ I_{DD}\left(t\right)\propto D^{*}\left(t\right) \end{array}$$

where D* 0 is the excited state concentration of the donor at *t* = 0. *k*_d_ is the rate constant of donor molecule de-excitation in the absence of acceptor and is equal to 1/τ_d_, in which τ_d_ is the donor fluorescence lifetime, and *k*_t_ is the rate constant of resonance energy transfer from donor to acceptor. The observed rate constant is *k*_d_ + *k*_t_ = *k*_da_. 1/*k*_da_ is equal to τ_da_, which is the fluorescence lifetime of the donor in the presence of the acceptor.

Similarly, the time dependence of the excited state concentration of the acceptor (A^*^(*t*)), generated via FRET, is directly proportional to the time-resolved fluorescence intensity *I*_DA_(*t*), in which the first subscript *D* denotes donor excitation wavelength and the second subscript *A* denotes acceptor emission wavelength:

(2) $$\begin{array}{l} A^{*}\left(t\right)=\frac{D_{0}^{*}k_{t}}{k_{t}+k_{t}-k_{a}}\left[e^{-k_{a}t}-e^{-\left(k_{d}+k_{t}\right)t}\right]\\ I_{DA}\left(t\right)\propto A^{*}\left(t\right) \end{array}$$

where A^*^(*t*) is the sum of two exponential components, of which one has a negative pre-exponential factor and the other has a positive pre-exponential factor (see details in [14]). The negative component reflects a rise of acceptor fluorescence due to energy transfer from donor to acceptor with rate constant *k*_d_ + *k*_t_ = *k*_da_, which is the same as the rate constant of the donor decay (Equation (1)) observed at the donor emission wavelength. 1/*k*_da_ is equal to τ_da_, which is the fluorescence lifetime of the donor in presence of the acceptor. The positive component arises from acceptor de-excitation with rate constant *k*_a_ and is equal to 1/τ_a_, in which τ_a_ is the acceptor fluorescence lifetime.

In the ideal case, the acceptor receives energy only from donor molecules via FRET, which implies that the absolute value of the ratio between negative (*A*_−_) and positive (*A*_+_) amplitudes is equal to 1. However, because of overlap between the absorption bands of the donor and acceptor, it is, in practice, impossible to selectively excite only donor fluorophores. The decay of acceptor fluorescence then contains an additional term with a positive amplitude, which arises from the detection of photons from directly excited acceptor molecules. Equation (2) must then be rewritten as:

(3) $$\begin{array}{l} A^{*}\left(t\right)=\left\{\frac{D_{0}^{*}k_{t}}{k_{d}+k_{t}-k_{a}}+A_{0}^{*}\right\}e^{-k_{a}t}-\frac{D_{0}^{*}k_{t}}{k_{d}+k_{t}-k_{a}}e^{-\left(k_{d}+k_{t}\right)t}\\ I_{DA}\left(t\right)\propto A^{*}\left(t\right) \end{array}$$

where A* 0 is the excited state concentration of directly excited acceptors at *t* = 0. Therefore, the absolute value of *A*_−_/*A*_+_ (|*A*_−_/*A*_+_|) of time-dependent acceptor fluorescence is usually smaller than 1.

An additional factor that results in the non-unity of the absolute ratio between negative and positive amplitudes is the detection of fluorescence photons from the donor in the acceptor detection window (*i.e.*, cross-talk) because of overlapping emission bands. These photons can come from both the FRET-active donors, as well as from FRET-inactive donor molecules that have no acceptors in their proximity. In the latter case, one has a heterogeneous system. The rate constants associated with the fluorescence decay of these FRET-inactive donor molecules might be similar to the decay rate of the acceptor, which makes it impossible to resolve them as separate decay components in the fluorescence signal detected in the acceptor window (A* 0 in Equation (3) is then composed of both donor and acceptor contributions). In addition, direct excitation of acceptor molecules also leads to a decrease of |*A*_−_/*A*_+_|. However, the exponential rate constant connected with the rise term in Equation (3) will not be affected, even if the absolute amplitude ratio is significantly smaller than unity. This is the main advantage of rise time measurements, since one can obtain the pure FRET rate constants in a heterogeneous donor-acceptor system.

The fluorescence lifetime of donor molecules in the presence of FRET is shorter than the one of the donor in the absence of an acceptor and can be calculated using:

(4) $$\tau_{da}=\frac{1}{k_{d}+k_{t}}$$

The fluorescence lifetime τ_da_ obtained from the rise time can be used to calculate the transfer rate constant:

(5) $$k_{t}=k_{da}-k_{d}=\frac{1}{\tau_{da}}-\frac{1}{\tau_{d}}$$

The transfer rate constant (*k*_t_) is proportional to the inverse sixth power of the distance *r*_da_ between donor and acceptor, which makes it a sensitive parameter for obtaining distances less than 10 nm:

(6) $$k_{t}=\frac{1}{\tau_{d}}{\left(\frac{R_{0}}{r_{da}}\right)}^{6}$$

where *R*_0_ is the so-called critical or Förster radius, the distance between donor and acceptor at which 50% of the donor energy is transferred to the acceptor. Through rearrangement of Equation (6), one obtains the following relationship:

(7) $$r_{da}=\frac{R_{0}}{{\left(\tau_{d}\cdot k_{t}\right)}^{1/6}}$$

### 3.2. Preparation of Double-Labeled Apoflavodoxin

Apoflavodoxin has a single, wild-type cysteine at position 69 [31,32]. Protein engineering and purification of the apoflavodoxin variant in which residue S178 is replaced by a cysteine (S178C) is described elsewhere [8]. Flavodoxin, *i.e.*, apoflavodoxin with the flavin mononucleotide cofactor (FMN), was first incubated with Alexa Fluor C5 568 maleimide (Invitrogen, Carlsbad, CA, USA) according to the protocol of the manufacturer in 100 mM potassium pyrophosphate (KPPi; Sigma-Aldrich, St. Louis, MO, USA), pH 7.5, in the presence of 1 mM Tris(2-carboxyethyl)phosphine (TCEP; Sigma, St Louis, MO, USA), for 1 h, at room temperature, in the dark. An excess of reduced glutathione was used to quench the maleimide moiety of the unreacted label, prior to unfolding of the protein by the addition of an equal volume of 7.5 M GuHCl. Upon unfolding, FMN is released from acceptor-labeled flavodoxin. A precast Biogel P6DG column (Bio-Rad, Hercules, CA, USA), equilibrated in 3.5 M GuHCl in KPPi, pH 7.5, was used to separate protein from FMN and unreacted label. To the fractions containing protein (identified as high molecular weight fractions with the color of the acceptor), 10× molar excess of Alexa Fluor 488 C5 maleimide was added, which was followed by incubation in the dark during 1 h. Subsequently, reduced glutathione was added to quench the unreacted maleimide, followed by the concentration of the protein using a Centricon spin filter with a cutoff of 10 kDa (Millipore, Billerica, MA, USA). The concentrated protein was refolded in 100 mM KPPi pH 6.0 on a Superdex 75 HR 10/30 column (GE Life Sciences, Buckingham, UK), and fractions containing monomeric double-labeled apoflavodoxin were snap-frozen in liquid nitrogen and stored at −80 °C until use.

### 3.3. Denaturant Dependent Equilibrium (Un)folding

The buffer used in all experiments with purified protein was 100 mM KPPi, pH 6.0, and contained 0.001% Tween-20 to prevent the adsorption of protein to surfaces. The temperature was set to 20 °C. A volume range of protein in 6.5 M GuHCl was added to a volume range of solutions of protein in buffer. Protein concentrations in both solutions were identical. After each addition, the sample was mixed and incubated for 5 min to reach equilibrium before measurement. A similar titration of solutions without protein was prepared to function as the background for time-resolved fluorescence.

### 3.4. Acquisition and Fitting of Time-Resolved Fluorescence Data

Picosecond-resolved fluorescence measurements were carried out using mode-locked continuous wave lasers for excitation and time-correlated single-photon counting (TCSPC) as the detection technique, as described previously [16,33]. The pulse duration was 0.2 ps; pulse energies were at the pJ level, and the repetition rate of pulses was 3.86 MHz. Decay curves were acquired by collecting photons in 4096 channels of a multi-channel analyzer using a channel time spacing of 5.0 ps. Laser pulses were vertically polarized, and fluorescence was detected via a polarizer oriented at the magic angle (54.7°) with respect to vertical polarization. The donor (A488) excitation wavelength was 450 nm, and the acceptor (A568) detection wavelength was 603 nm, using a Schott interference filter (11.9-nm bandwidth; Schott, Mainz, Germany). Samples without protein were used to determine background fluorescence under identical experimental conditions. The dynamic instrumental response function (*IRF*) was determined using a freshly made solution of erythrosine B in water as reference compound, which has a fluorescence lifetime τ_ref_ = 89 ps at 20 °C [34]. Fluorescence photons were collected at a frequency of about 30 kHz. We used acquisition times of 200, 40 and 60 s for protein samples, background and reference, respectively. This resulted in about 6000 counts in the peak for the protein samples and about 29,000 counts in the peak for the reference compound.

Fluorescence rise and decay curves were analyzed using the TRFA data processor (Scientific Software Technologies Center, Minsk, Belarus; www.sstcenter.com). Each of the decay curves is described by a bi-exponential decay function (Equation (3)) that was convolved with the *IRF*. Because of the finite width of the *IRF*, Equation (3) is rewritten as:

(8) $$i_{DA}\left(t\right)=I_{DA}\left(t\right)⊗IRF\left(t\right)$$

where *i*_DA_(*t*) is the experimental time-dependent acceptor fluorescence, *I_DA_*(*t*) is the model function as given by Equation (3) and is the convolution operator. Equation (8) forms the basis of the reference convolution method, of which details are given in [35]. Two thousand time channels were used in the analysis. Confidence limits of time constants and amplitudes were determined by a rigorous error analysis at the 67% confidence level. The quality of the fits was judged by the χ^2^ criterion, which should be equal to 1 for an optimal fit. In addition, the weighted residuals were used for the visual inspection of the agreement between measured (*y*_exp_) and model-generated (*y*_calc_) data. The weighted residuals (*WR*) in each time point *k* were calculated as:

(9) $$WR\left(k\right)=\frac{\left[y_{exp}\left(k\right)-y_{calc}\left(k\right)\right]}{\sigma_{k}}=\frac{\left[y_{exp}\left(k\right)-y_{calc}\left(k\right)\right]}{\sqrt{y_{exp}\left(k\right)}}$$

where *k* = 1, …, 2000 and σ is the standard deviation.

Fluorescence decay data of donor only protein (in which donor A488 is linked to Cys69 of apoflavodoxin; A488-apoflavodoxin, excitation wavelength at 450 nm and detection wavelength at 512.2 nm) have been reported previously [8].

## 4. Conclusions

Determination of acceptor fluorescence rise times enables quantification of FRET between donor and acceptor molecules attached to a protein of interest, yielding inter-dye distances. We demonstrate that this spectroscopic approach tracks protein folding. The key advantage of this methodology is that one only monitors those protein molecules that actually exhibit FRET between donor and acceptor, because FRET-inactive donor populations do not contaminate the measured rise and decay times of acceptor fluorescence.

## Supplementary Materials

Supplementary materials can be found at: http://www.mdpi.com/1422-0067/15/12/23836/s1.
